# Optimization of the Production of *ε*-Poly-L-Lysine by Novel Producer Lactic Acid Bacteria Isolated from Traditional Dairy Products

**DOI:** 10.1155/2020/2145656

**Published:** 2020-10-05

**Authors:** Hamid Reza Samadlouie, Shahrokh Gharanjik, Zohreh Beygom Tabatabaie

**Affiliations:** ^1^Department of Food Science and Technology, Faculty of Agriculture, Shahrood University of Technology, Shahrood, Iran; ^2^Department of Plant Breeding and Biotechnology, Faculty of Agricultural Engineering, Shahrood University of Technology, Shahrood, Iran

## Abstract

New strains of lactic acid bacteria (LAB) were isolated from different traditional dairy products. Six new strains named *Lactobacillus delbrueckii* strain A01, *Lactobacillus delbrueckii* subsp. *bulgaricus* strain D01, *Lactobacillus delbrueckii* subsp. *bulgaricus* strain E01, *Lactococcus lactis* strain G01, *Lactobacillus delbrueckii* strain C01, and *Lactobacillus delbrueckii* subsp. *bulgaricus* strain F01 were identified using 16S rDNA sequencing, morphological and biochemical traits. All strains have been registered in the National Center for Biotechnology Information (NCBI) with accession numbers MN611241.1, MN611300.1, MN611301.1, MN611303.1, MN611241.1, and MN611299.1, respectively. Having found *ε*-Poly-L-Lysine (*ε*-PL) in all strains isolated, *Lactobacillus delbrueckii* strain A01 was identified as an active producer of *ε*-Poly-L-Lysine (*ε*-PL). The one-factor-at-a-time method and central composite design were applied to optimize *ε*-Poly-L-Lysine (*ε*-PL). A predicted 200 ppm of *ε*-PL was obtained in the medium containing the lowest level of glucose, 25 g/l, and yeast extract, 6 g/l.

## 1. Introduction

Lactic acid bacteria impart curative properties and antimicrobial activities to dairy products [1]. Lactic acid bacteria (LAB) with probiotic characteristics have considerable beneficial health effects on the intestinal surface once introduced in adequate amounts [2, 3]. New research indicated that synbiotic yoghurt containing *Lactobacillus acidophilus* reduced the cholesterol and triglycerides of rabbit blood [4]. Besides these outstanding features, LAB confers many benefits such as boosting the immune system and reducing the incidence and severity of diseases [5]. For instance, *Lactobacillus acidophilus* had a detrimental influence on foodborne pathogens like *Escherichia coli* and *Staphylococcus aureus* [6]. These characteristics make the strains an apt choice for natural food and feed preservatives [5]. With regard to the abovementioned remarkable characteristics of LAB, there have been many efforts to identify LAB in a variety of natural habitats [7, 8]. Among various dairy beverages, traditional doogh is a local fermented product in Iran [9, 10]. Various research studies have employed phenotypic characteristics and biochemical properties to identify LAB in dairy products [11], while a PCR-based technique was a more effective way to precisely identify LBA than the conventional methods. Using this reliable method has led to the discovery of various microorganisms all across the world [12]. Various bioactive components produced by LAB could be stimulated by acidic media with detrimental effects on the putrefactive bacterial growth and exert a modifying and controlling influence on the beneficial microbial gastrointestinal tract [13, 14]. *ε*-PL as secondary metabolism has created great attention among scientists who want to analyze their curative characteristics. Scientists have discovered that the family of Streptomycetaceae has had good potential for *ε*-PL production ([15]; Shoji [16]) with various health beneficial properties, especially antimicrobial activities (M. [17]; Shoji [18, 19]). *ε*-PL improves physiochemical properties and shelf life of food products. Besides the wide antimicrobial spectrum property of *ε*-PL, it was also recognized as drug delivery with antitumor and antioxidant properties [20, 21]. One of the primary objectives of milk fermentation using LAB is to extend the shelf life of dairy products. Possibly one of the main reasons why fermented dairy products have had a long shelf life is because of the active components with antimicrobial properties, given that initial experiments conducted in this research revealed that the isolated LAB turned out to be good sources of *ε*-PL. Therefore, the main aim of this research was to isolate and identify LAB in traditional dairy products by genetic, morphological, and biochemical methods. Finally, the strain with the highest potential for producing *ε*-PL was selected for optimization by using the one-factor-at-a time and RSM methods.

## 2. Materials and Methods

### 2.1. Sample Collections

Traditional fermented milk, doogh and yoghurt, were collected randomly from rural areas, namely, Mojen, Kalatekhij, Kalposh, and near Shahrood in Iran. All samples were prepared under sanitary conditions. LAB was isolated and enumerated using MRS. All materials were also bought from Merck (Germany).

### 2.2. Isolation of LAB and Maintenance Method

All samples were diluted (1 : 10) with physiological water (0.85% NaCl). They were diluted by 7-fold serial dilutions. Then, 0.1 ml of the dilutions were mixed with melted MRS agar and incubated at 37°C for 24-48 h in anaerobic jars with 5% CO_2_; thereby, all possible LAB in each sample were identified with cell morphological features. All colonies in the bottom or middle of MRS agar were removed and being cultivated in MRS broth at 30°C for 72 h [22].

### 2.3. Phenotypic and Genotypic Identifications

Colony morphology was initially used to recognize the different types of LAB; thereby, bacterial shapes colored by gram staining were microscopically identified. Furthermore, catalase and cytochrome oxidase tests were carried out to assess the possible presence of LAB [23]. Biochemical and genetic analyses were conducted when gram-positive and catalase-negative bacteria composed of cocci and bacilli were isolated.

### 2.4. Biochemical Characteristics of Isolated Strains

10 types of carbohydrates (glucose, raffinose, mannitol, fructose, sucrose, starch, galactose, mannitol, sorbitol, and lactose) were used to examine the growth and acid and gas productions [24, 25].

### 2.5. DNA Extraction

The CinnaPure DNA kit was used to extract DNA from LAB. After purification of DNA, NanoDrop 2000 was used to assess the quality and quantity of DNA. Two universal LAB primers 27F (forward: 5′-AGAGTTTGATCCTGGCTCAG-3′) and 1492R (reverse: 5′-CTACGGCTACCTTGTTACGA-3′) were used to amplify 16S rRNA under the following conditions: 5 min at 95 C, 33 cycles of 30 s at 94 C, 30 s at 54 C, 2 min at 72 C, and a final extension at 72 C for 10 min [26]. Then, all extracted DNA were sent for DNA sequencing to Takapouzist Company (http://www.takapouzist.com). Codon Code Aligner and MEGA5 were used for conducting sequence alignment, inferring phylogenetic trees. The clustering method UPGMA was used to draw the phylogeny with the aid of the MEGA 6.0 1000 bootstrap replicates. The consensus trees were also drawn by MEGA 6.0. The results attained from the genotypic identifications were compared to verify coinciding results, using the biochemical and molecular identification.

### 2.6. *ε*-PL Assessment

0.02% of methylene blue as an indicator was added to the medium containing (g/l) glycerol 10, ammonium sulfate 1.0, disodium hydrogen phosphate 0.5, magnesium sulfate 0.25, yeast extract 0.5, potassium dihydrogen phosphate 0.5, and agar 2%, at pH 7. All samples were streaked on the formulated medium and incubated at 37°c for 168 h. The outer zone indicates the ability of the strain to produce *ε*-PL [27].

### 2.7. *ε*-PL Assay

Once the cultures entered the stationary phase, cells were centrifuged (9000 rpm, 10 min) and the supernatant was used to assess the *ε*-PL concentration.

Selective binding of trypan blue as an indicator to *ε*-PL leads to a reduction of the indicator color, recognized as a sensitive and selective method to detect *ε*-PL. 20.88 ml of 0.1 mM phosphate buffer (pH 7.0) containing 120 *μ*l of trypan blue solution (1 mg/ml) was mixed thoroughly with 1 ml of the supernatant, then placed in a hot water bath at 37°c for 60 min. A UV-vis spectrophotometer was used to detect the color reduction at 580 nm. The standard of *ε*-PL was serially diluted to 0-50 mg/l dilution used to derive an equation between color reduction and *ε*-PL content which is termed as the standard curve [27].

### 2.8. Optimization of Media for *ε*-PL

Based on research studies done on various strains for *ε*-PL production, various carbon and nitrogen sources were selected to identify the key substrates using the one-factor-at-a-time method; then, various concentrations of key substrates as variable factors were optimized by the RSM method; (NH_4_)_2_SO4 as organic nitrogen was also used [28]. Among various strains, the species with high efficiency to reduce the blue color of trypan blue in the solid defined medium was selected as a highly active *ε*-PL-producing strain.

## 3. Result and Discussion

### 3.1. LAB in Kalatekhij Doogh

Three distinct colony gram-positive and catalase-negative bacilli, isolated from Kalatekhij doogh termed as A01, D01, and E01, were enumerated on MRS agar at 37°C after 72 h. 6 × 10^7^ CFU ml^−1^ A01, 5 × 10^7^ CFU ml^−1^ D01, and 5 × 10^8^ CFU ml^−1^ E01 were counted.

#### 3.1.1. Genetic Method for Identification of Isolated Strains Present in Kalatekhij Doogh

The mentioned strains were incubated in the liquid MRS medium at 37°C. DNA were extracted on the basis of kit instruction. NanoDrop was used to determine the quality and quantity of extracted DNA.

**Figure 1:**
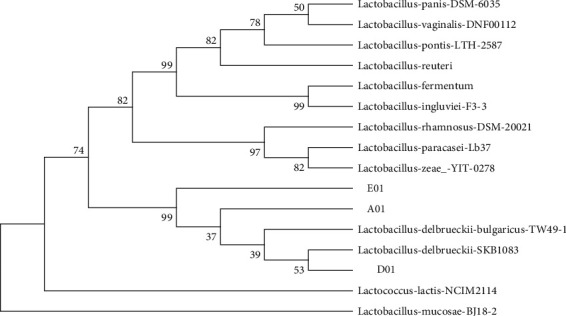
Phylogenetic tree of the unknown isolates of lactic acid bacteria with 13 similar species based on nearest neighbor interchange analysis of 16S rDNA (bootstrap level: 1000 pseudoreplications).

The phylogenetic tree indicated that all strains were so similar to *Lactobacillus delbrueckii*. The species were initially determined by the BLAST program on NCBI (https://www.ncbi.nlm.nih.gov/nuccore/MN611303.1) with more than 99% similarity with reference strains. Then, all isolates and related reference strains were considered to construct the phylogenetic tree (Figure 1).

#### 3.1.2. Biochemical Assessments of the Isolated LAB in Various Carbon Sources

Acid and gas productions were studied over various carbon sources like glucose, sucrose, raffinose, maltose, fructose, lactose, mannitol, galactose, sorbitol, and starch. Acid and gas were produced by all isolated LAB in all carbon sources except for the starch substrate, so the strains were considered to be homofermentative. Based on the phylogenetic tree and initial biochemical and morphological tests, the isolated species were named as *Lactobacillus delbrueckii* strain A01, *Lactobacillus delbrueckii* subsp. *bulgaricus* strain D01, and *Lactobacillus delbrueckii* subsp. *bulgaricus* strain E01, with the GenBank accession numbers of MN611241.1, MN611300.1, and MN611301.1, respectively.

### 3.2. Investigation of the Isolated LAB in Kalposh Doogh

**Figure 2:**
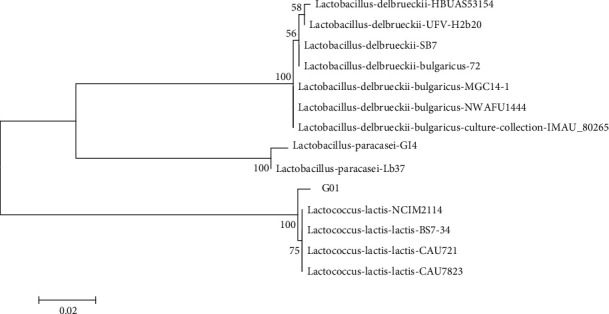
Phylogenetic tree of the unknown isolates of lactic acid bacteria with 13 similar species based on nearest neighbor interchange analysis of 16S rDNA (bootstrap level: 1000 pseudoreplications).

A gram-positive and catalase-negative coccus found in Kalposh doogh at an average number of 5 × 10^7^ CFU ml^−1^ was named G1. The nucleotide sequence was used to construct a phylogenetic tree using 13 different ribosomal RNA gene sequences. The result indicated that the strain was so similar to *Lactococcus lactis* (Figure 2).

#### 3.2.1. Biochemical Test of Isolated Strain

In all aforementioned carbon sources, the strain could grow without gas productions. The strain was able to produce acid in all samples except for sorbitol and starch. With regard to the biochemical and genetic results, this strain was named *Lactococcus lactis* G01 with the GenBank accession numbers of MN611303.1.

### 3.3. Isolation of LAB from Kalatekhij Yoghurt

Kalatekhij yoghurt was used to isolate LAB. Two distinct colony morphologies were detected and named as C01 and F01. 2 × 10^8^ CFU ml^−1^ C01 and 1 × 10^8^ CFU ml^−1^ F01 were counted in the yoghurt sample. The initial tests indicated that both species were gram-positive and catalase-negative bacilli.

**Figure 3:**
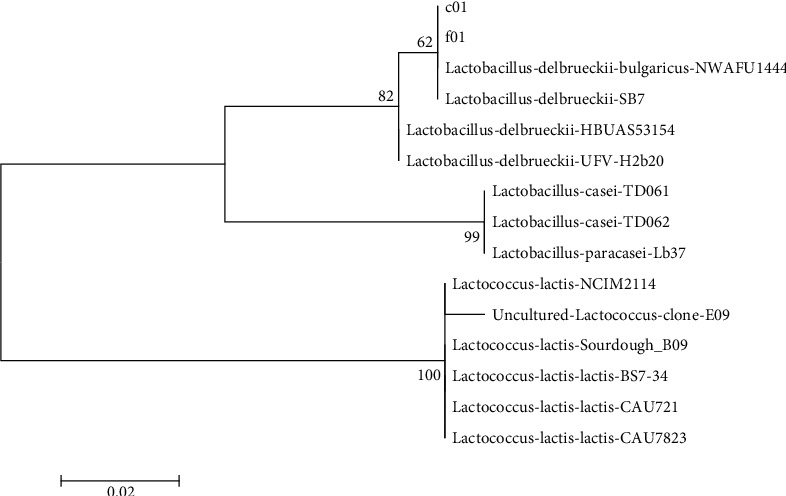
Phylogenetic tree of the unknown isolates of lactic acid bacteria with 13 similar species based on nearest neighbor interchange analysis of 16S rDNA (bootstrap level: 1000 pseudoreplications).

DNA sequence indicated that C01 and F01 were so similar to *Lactobacillus delbrueckii*. (Figure 3). To be more precise, C01 and F01 were named *Lactobacillus delbrueckii* strain C01 and *Lactobacillus delbrueckii* subsp. *bulgaricus* strain F01 which were registered in the NCBI site with the accession numbers MN611241.1 and MN611299.1, respectively.

### 3.4. *ε*-PL Production

A polymer called *ε*-PL is made by the condensation reaction of 25-35 L-lysine residues. This homopolymer appears to be a strong antimicrobial agent against vast groups of microorganisms. *ε*-PL as a naturally effective food preservative has attracted great interest from researchers who want to assess new microbial resources of such preservation [29]. Such a property makes it an ideal component for various applications and uses in the food and pharmaceutical industries.

There are several ways to assess *ε*-PL in the media. Anionic dye as a color indicator undergoes color changes once it bonded to *ε*-PL in which the changes are precisely detected by spectrophotometry [30]. Shen et al. [31] stated that using trypan blue with the threshold of detection of 1-10 *μ*g *ε*-PL makes the method more accurate than other spectrophotometric methods. So in the present research, all isolates were first cultivated in the plates containing trypan blue to initially examine the presence of *ε*-PL. Among all isolated samples, *Lactobacillus delbrueckii* strain A01 was the most active producer of *ε*-PL. Regarding the important role of *ε*-PL in food and pharmacological industries, *Lactobacillus delbrueckii* strain A01 was used to optimize *ε*-PL production under the given conditions designed by the statistical approaches one-factor-at-a-time method and RSM.

#### 3.4.1. Carbon Sources

**Figure 4:**
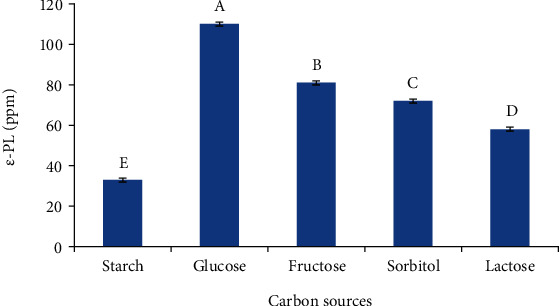
Effect of various carbon sources on *ε*-PL production.

Temperature (37°C), yeast extract (10 g/l), stirring round (110 rpm), and 3 days of fermentation were constant factors. On the other hand, carbon sources (starch, glucose, fructose, sorbitol, and lactose) were variable factors. Results indicated that carbon sources exerted high influences on *ε*-PL productions as can been seen in Figure 4. Glucose supported the highest content of *ε*-PL production which stood at 112 ppm in the formulated medium, followed by fructose, sorbitol, and lactose, respectively. By contrast, the lowest content of *ε*-PL was attained in the medium containing starch as carbon sources (Figure 4). This result indicated that the isolated strain has the lowest capability to degrade and assimilate starch as carbon sources. Various research studies declared that glucose was a good source of carbon which supported the highest content of *ε*-PL [32].

#### 3.4.2. Protein Sources

Temperature (37°C), glucose (20 g/l), and rotational speed at 170 rpm for 3 days were regarded as the constant factors of the fermentation conditions. Under this condition, the effects of various nitrogen sources on *ε*-PL production were investigated.

**Figure 5:**
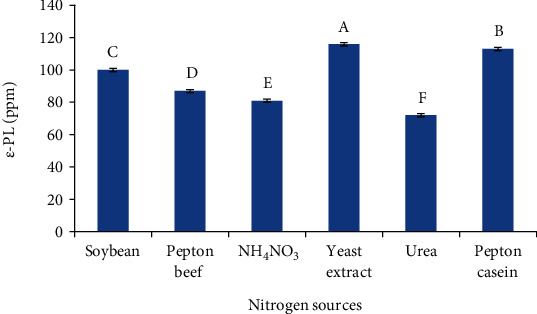
Effect of various nitrogen resources on *ε*-PL production.

As can be seen in Figure 5, yeast extract was by far the best medium stimulating *ε*-PL production, followed closely by peptone casein and soybean media. By contrast, the least amounts of *ε*-PL were attained in media containing inorganic nitrogen sources like NH_4_NO_3_ and urea. In line with these results, the research study indicated that among different protein sources examined, yeast extract seemed to be more effective in *ε*-PL yield compared to the other protein sources (Fengzhu [33]) so glucose and yeast extract were apt choices for *ε*-PL production.

Various researchers claimed that substrate compositions mainly affected the quantity of *ε*-PL production. The carbon skeletons of amino acids as the main precursor of *ε*-PL come from intermediates of the glycolysis pathway. Glucose as a major carbohydrate is utilized within the glycolysis pathway; as a result, such carbon sources stimulate more *ε*-PL production. More to the point, lysine was polymerized by peptide bonds to form *ε*-PL [32]. Markedly, of all substrates used for *ε*-PL induction, adequate amounts of glucose and lysine seem more important (S [34]).

### 3.5. Optimization of *ε*-PL Production Using RSM

Optimization designs of experiments provide a mathematical model for predicting the process behavior. Response surface methodology (RSM) requires fewer experiments than conventional designs [35], to optimize a specific response impacted by variables. The main objective of this research was to increase *ε*-PL production, so a defined range of two key substrates carbon source (glucose) and nitrogen source (yeast extract) was formulated according to RSM (Table 1). Aerobic fermentation has been used to trigger *ε*-PL. *ε*-PL production was stimulated in the stationary phase at low pH around 3-5 (Shoji [15, 36]). Based on the aforementioned studies, rotational speed at 170 rpm and pH 5 served as stimulating factors. As can be seen in Table 2, statistic results indicated that the linear effects of yeast extract and glucose on the amount of *ε*-PL were significant (*p* < 0.0001). The quadratic effect of glucose and yeast extract on the production of *ε*-PL was significant (*p* < 0.0021 and 0.0003). The effect of glucose-yeast extract interaction on *ε*-PL was also significant (*p* < 0.014). The value of the coefficient of determination (*R*^2^) for the *ε*-PL formula was 0.99 which indicated the degree of conformity of the data in the regression model; as a result, it can be concluded that the regression model was well able to show and predict the relationship between the culture conditions (glucose and yeast extract) and *ε*-PL production.

As shown in Table 2, the *F* value relevant to yeast extract sources was higher than that of glucose; such a result indicated yeast extract, in comparison, has a significantly higher effect on *ε*-PL production. The central composite design of the response level method with the actual amount of data is reported in Table 1. As shown in Table 1, there had been a reverse correlation between the substrates, yeast extract and glucose content, and *ε*-PL production. Once glucose concentration declined to 25 g/l along with the high and constant level of yeast extract; *ε*-PL production remarkably climbed from 103.2 up to 131.10 (runs 4 and 1). Similar patterns were repeated at the lower rate for the yeast extract substrate (runs 7 and 8).

The fitted equation of *ε*-PL production over the glucose and yeast extract was shown, where *Y* was the *ε*-PL production and *A* and *B* were the glucose and yeast extract, respectively. The model terms with “Prob > *p*” less than 0.05 are regarded as significant: *P* = (+314.25985 + 0.59176 *A* − 29.06066 *B* + 0.075160*A* × *B* − 0.02393*A*^2^ + 0.98571 × *B*^2^).

#### 3.5.1. The Result of *ε*-PL Productions

**Figure 6:**
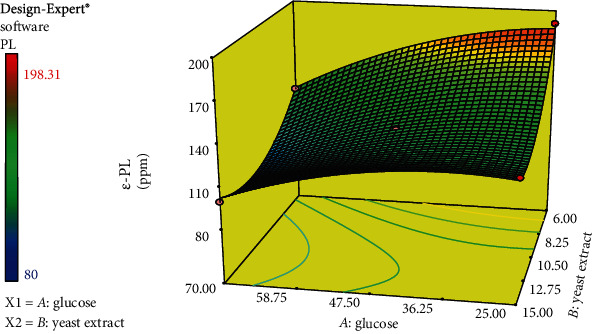
RSM curve for *ε*-PL production (ppm) by *Lactobacillus delbrueckii* strain A01. *A*, glucose; *B*, yeast extract.

Figure 6 shows the effect of various levels of glucose and yeast extract on *ε*-PL. The properties of the culture media have a significant impact on the production of *ε*-PL. The response surface indicated that there was a negative correlation between response and substrate contents. To be more precise, the highest amount of *ε*-PL was achieved in the medium containing the lowest level of glucose 25 g/l and yeast extract 6 g/l.

#### 3.5.2. The Predicted Optimum Levels of Substrates for *ε*-PL

The optimum content of the response, 186.5 ppm, was predicted by Design-Expert at 25 g/l glucose (*A*) and 6 g/l yeast extract (*B*), whereas the minimum predicted *ε*-PL, namely, 94.75 ppm, was obtained in the medium containing 70 g/l glucose and 12.7 g/l yeast extract. This result was in line with Saimura et al. [37] who stated that the excessive amount of lysine exerts an inhibitory influence on *ε*-PL production [37]. To evaluate the model, the predicted conditions of the *ε*-PL production model were applied. 25 glucose and 6 g/l yeast extract were used, and the initial pH was set at 5. After 3 days of fermentation, the massive 200 ppm of *ε*-PL was obtained, which is well close to the predicted value of the formulated medium. Importantly, Streptomycetaceae and Ergot fungi are the main organisms known to produce *ε*-PL [38]. 948 and 1085 g/l ppm *ε*-PL were optimized using RSM by *Streptomyces diastatochromogenes* and *Streptomyces albus* Y07, respectively (M. [19]) [39], while *Streptomyces violaceusniger* in optimal condition was able to produce a mere 349 ppm *ε*-PL [40], considerably lower than that of the two aforementioned *Streptomyces*. Obviously, 200 ppm *ε*-PL produced by *Lactobacillus delbrueckii* strain A01 indicated that the species could be considered a promising source of *ε*-PL.

## 4. Conclusion

The first attempt has been made to isolate and characterize LAB from the traditional fermented milk of the rural area near Shahrood in Iran. Six strains of LAB isolated from dairy products were identified and published with GenBank accession numbers. The result indicated that isolated strains named *Lactobacillus delbrueckii* strain A01, in optimal condition, could be regarded as a promising source of *ε*-PL. The highest content of *ε*-PL was attained in the medium containing the lowest content of protein and glucose. To conclude, a preserving activity of LAB in fermented dairy products could be somehow related to the *ε*-PL produced by the wild LAB in traditional dairy products.

## Figures and Tables

**Table 1:** Results of FCCCD using two variables indicating observed and predicted results.

Run	Glucose (g/l)	Yeast extract (g/l)	*ε*-PL (ppm)	
			Observed	Predict
1	-1 (25)	1 (15)	129.78	131.10
2	0 (47.5)	0 (10.5)	129.78	128.15
3	-1 (25)	-1 (6)	190	186.49
4	1 (70)	1 (15)	100	103.2
5	1.41 (79.32)	0 (10.5)	80	76.74
6	0 (47.5)	-1.41 (4.58)	198.31	199.79
7	0 (47.5)	0 (10.5)	129	132.1
8	0 (47.5)	1.41 (16.86)	140	138.82
9	1 (70)	-1 (6)	129.78	129.78
10	-1.41 (15.6)	0 (10.5)	130	133.56

**Table 2:** ANOVA parameters of the models fitted for *ε*-PL.

Source	Sum of squares	df	Mean square	*F* value	*p* value Prob > *F*	
Model	11392.37	5	2278.47	168.43	<0.0001	Significant
*A*-glucose	3228.49	1	3228.49	238.66	0.0001	
*B*-yeast extract	3717.93	1	3717.93	274.84	<0.0001	
*AB*	231.65	1	231.65	17.12	0.0144	
*A* ^2^	671.171	1	671.17	49.61	0.0021	
*B* ^2^	1821.38	1	1821.38	134.64	0.0003	
Residual	54.11	4	13.53			
Lack of fit	53.81	3	17.93	58.96	0.0954	Not significant
Pure error	0.30	1	0.30			
Cor total	11446.48	9				

## Data Availability

The original data used to support the findings of this study are included within the article.
